# Supplementary figures and images for: Chemical and spectroscopic characterization of (Artemisinin/Querctin/ Zinc) novel mixed ligand complex with assessment of its potent high antiviral activity against SARS-CoV-2 and antioxidant capacity against toxicity induced by acrylamide in male rats
Source: PeerJ. 2024 Jan 2;12:e15638. doi: 10.7717/peerj.15638 (PMC10768679; doi:10.7717/peerj.15638)

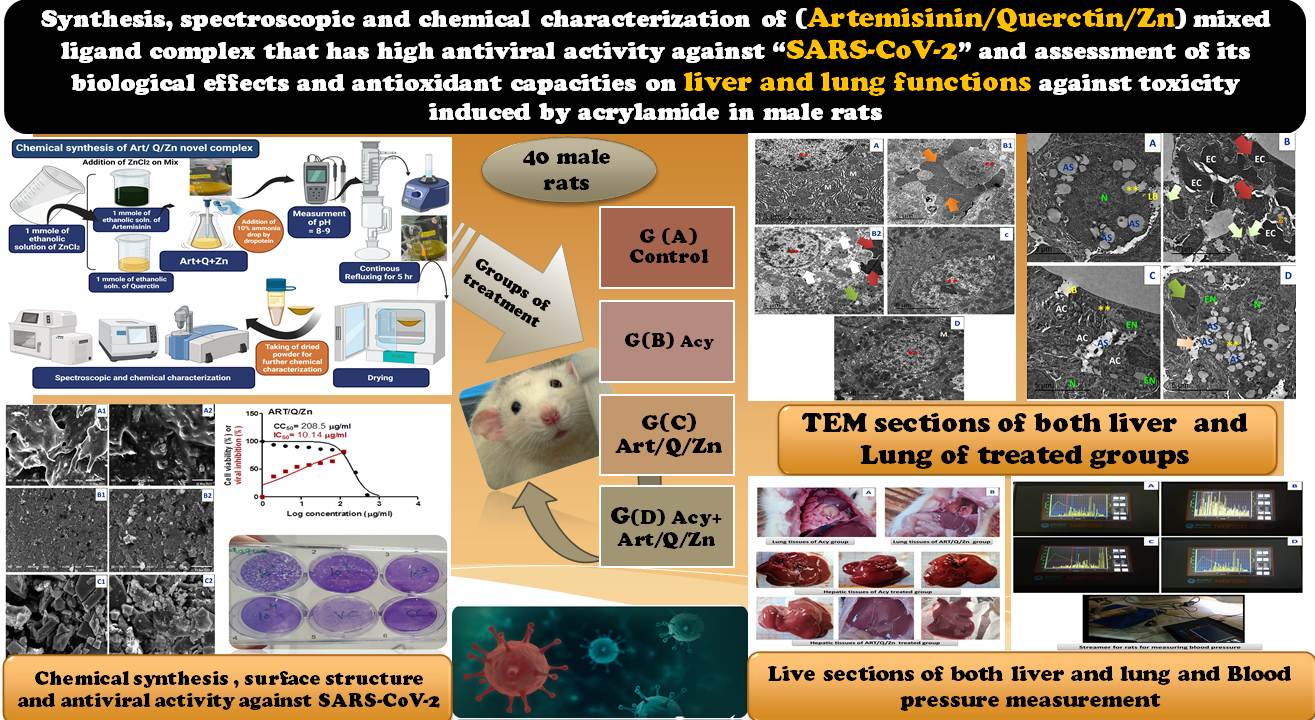

Supplement: Supplemental Information 1 [file peerj-12-15638-s001.jpg]

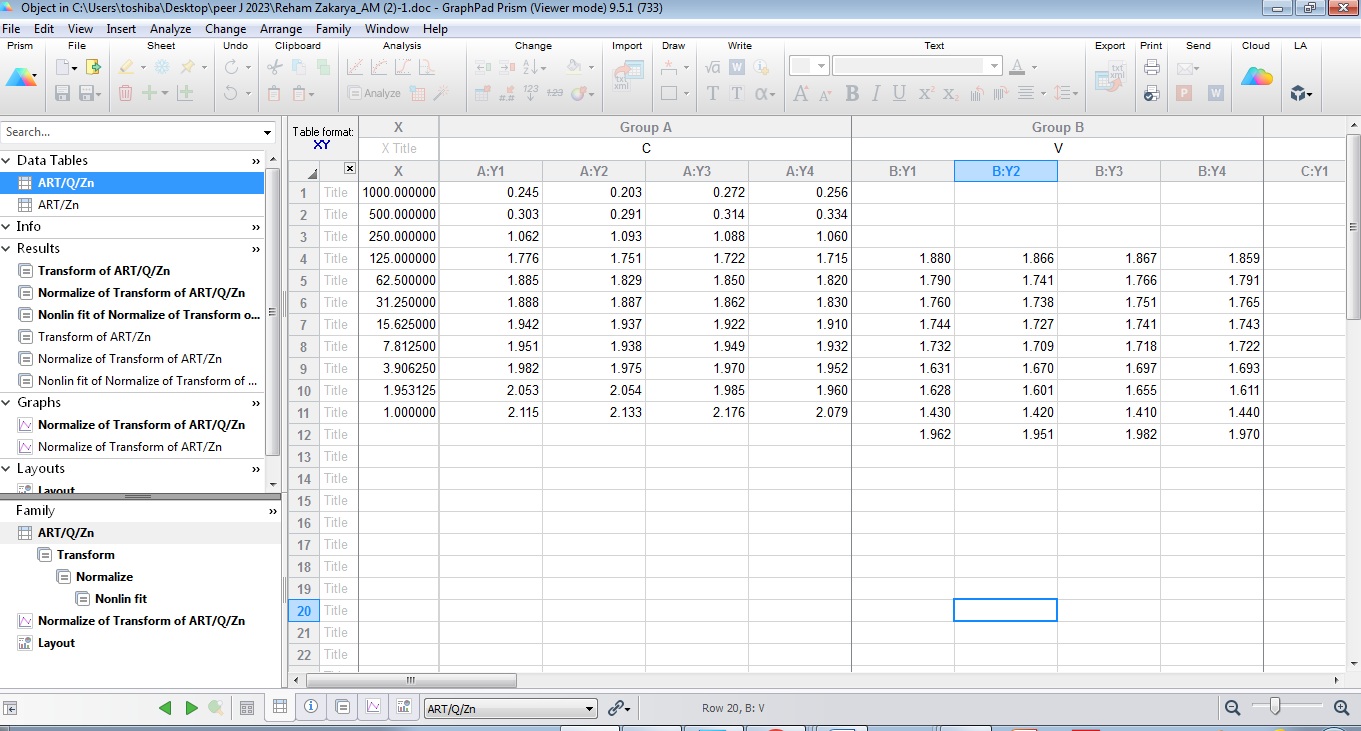

Supplement: Supplemental Information 2 [file peerj-12-15638-s002.jpg]

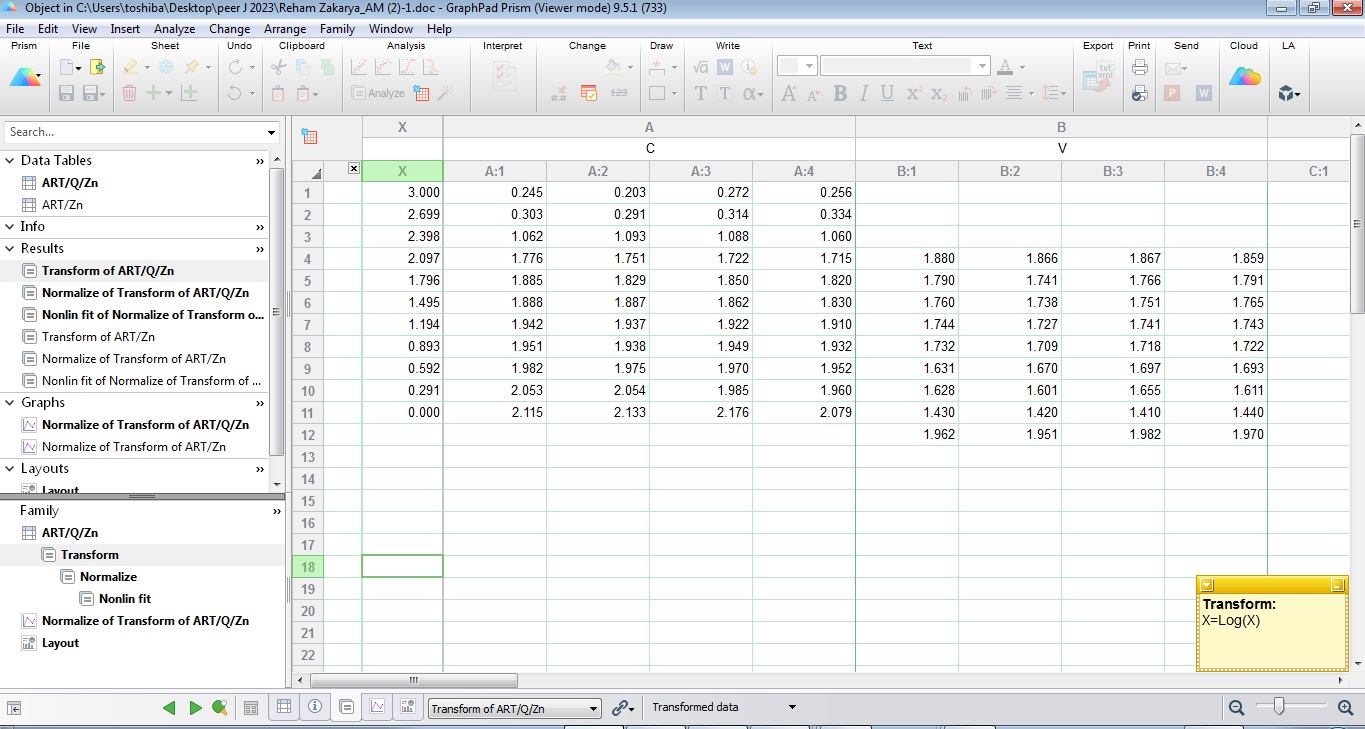

Supplement: Supplemental Information 3 [file peerj-12-15638-s003.jpg]

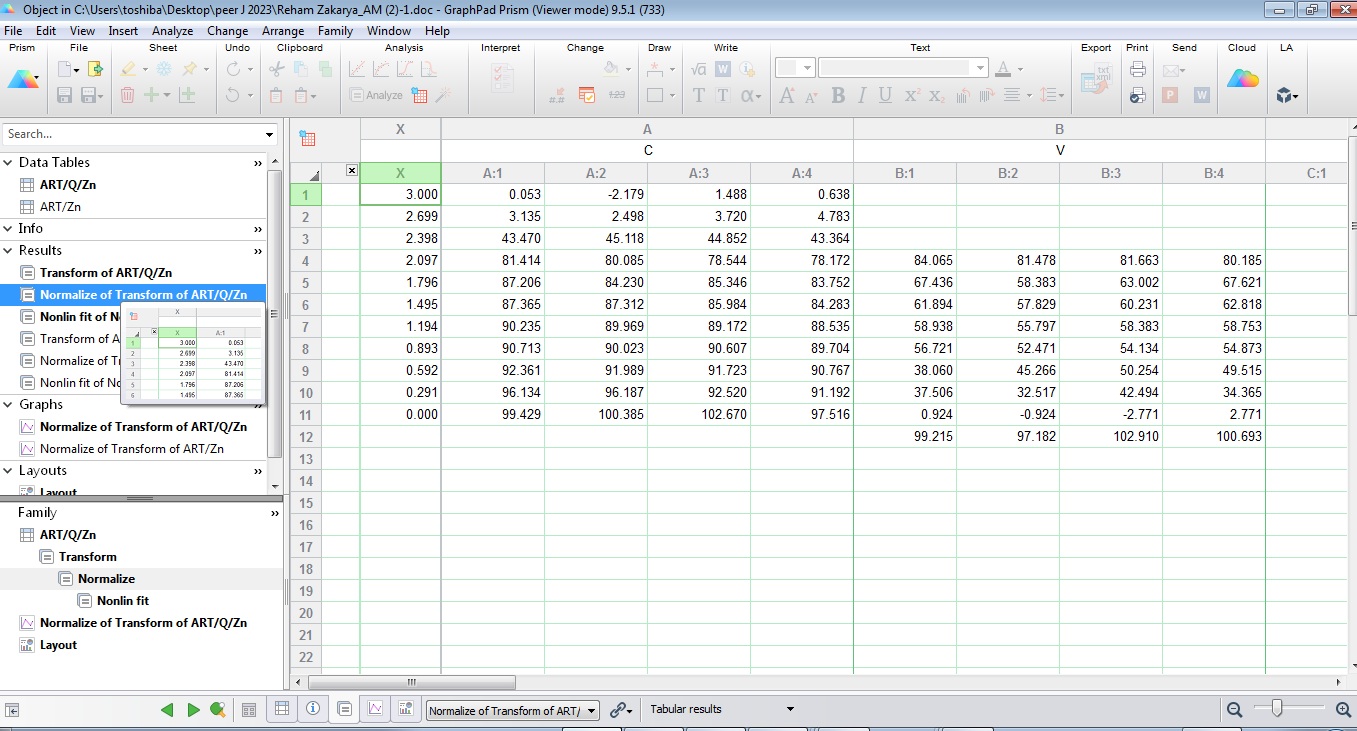

Supplement: Supplemental Information 4 [file peerj-12-15638-s004.jpg]

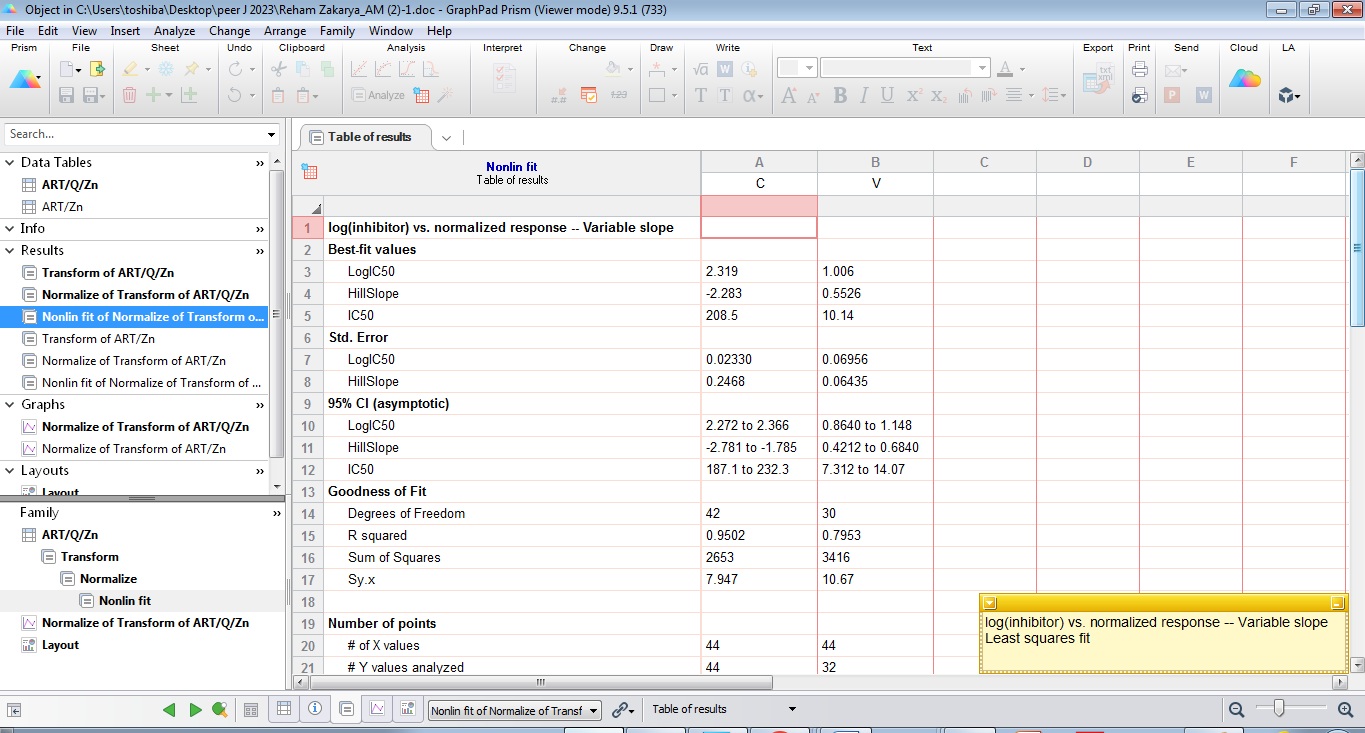

Supplement: Supplemental Information 5 [file peerj-12-15638-s005.jpg]

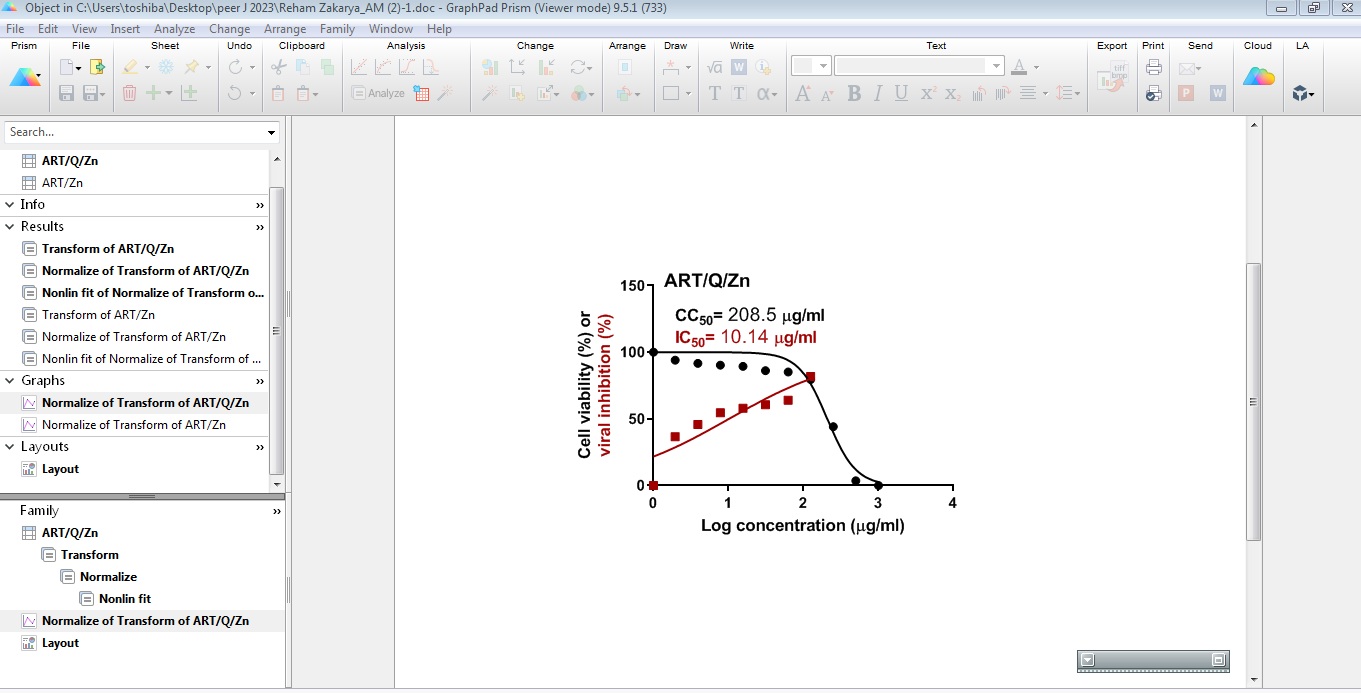

Supplement: Supplemental Information 6 [file peerj-12-15638-s006.jpg]

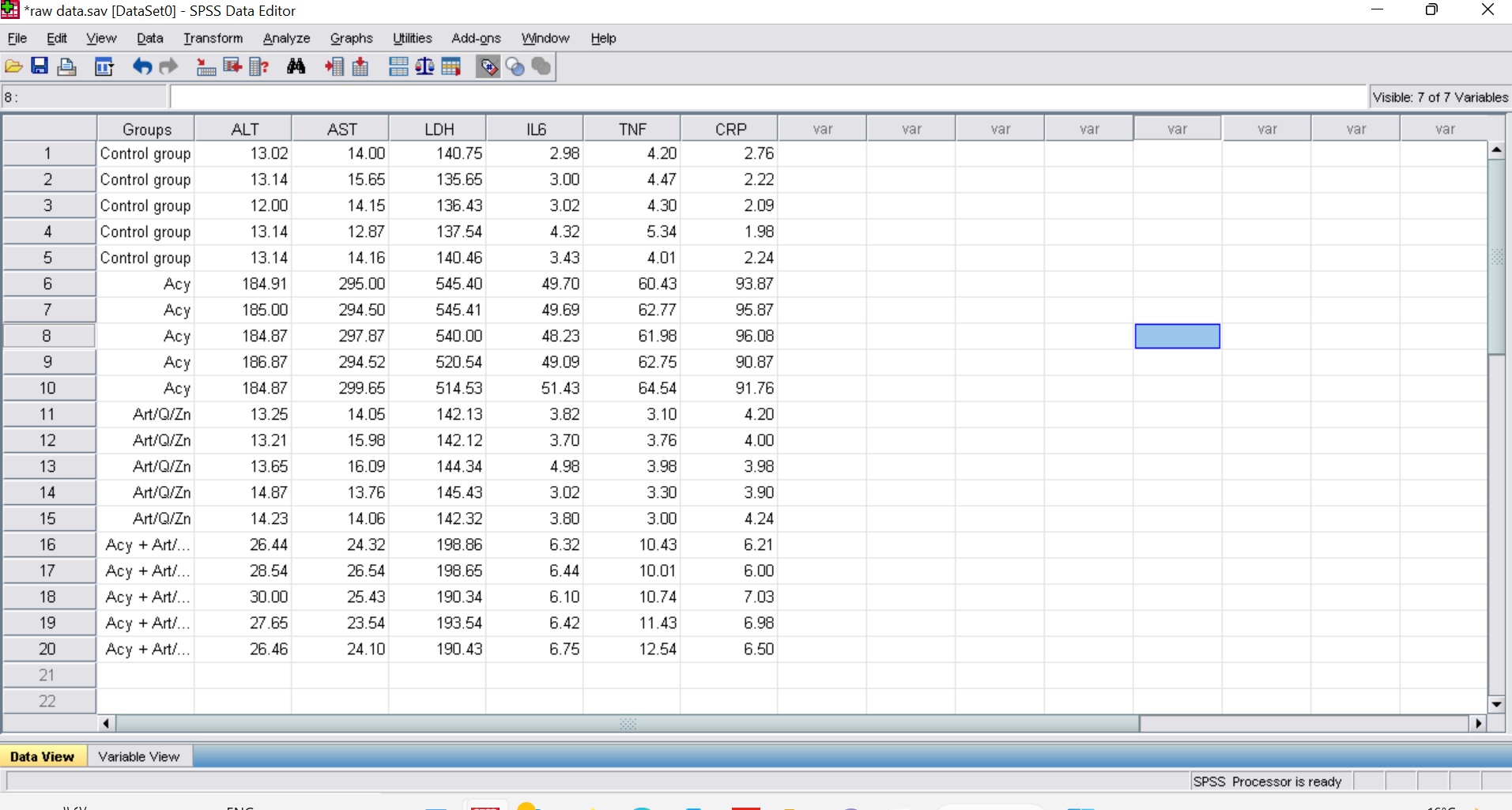

Supplement: Supplemental Information 7 [file peerj-12-15638-s007.jpg]

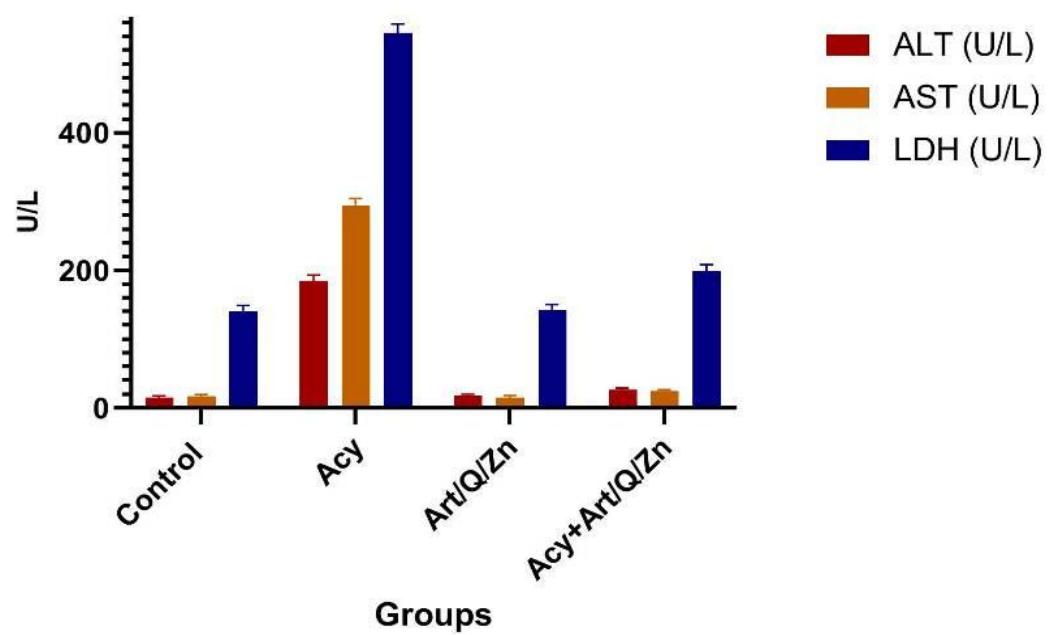

Supplement: Supplemental Information 11 [file peerj-12-15638-s011.pdf]

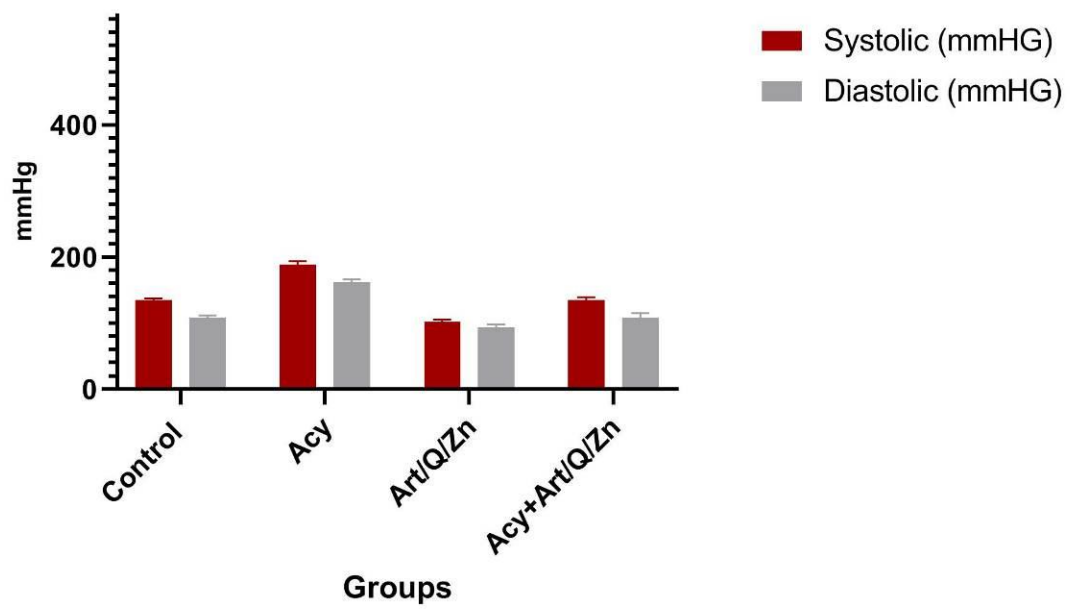

Supplement: Supplemental Information 12 [file peerj-12-15638-s012.pdf]

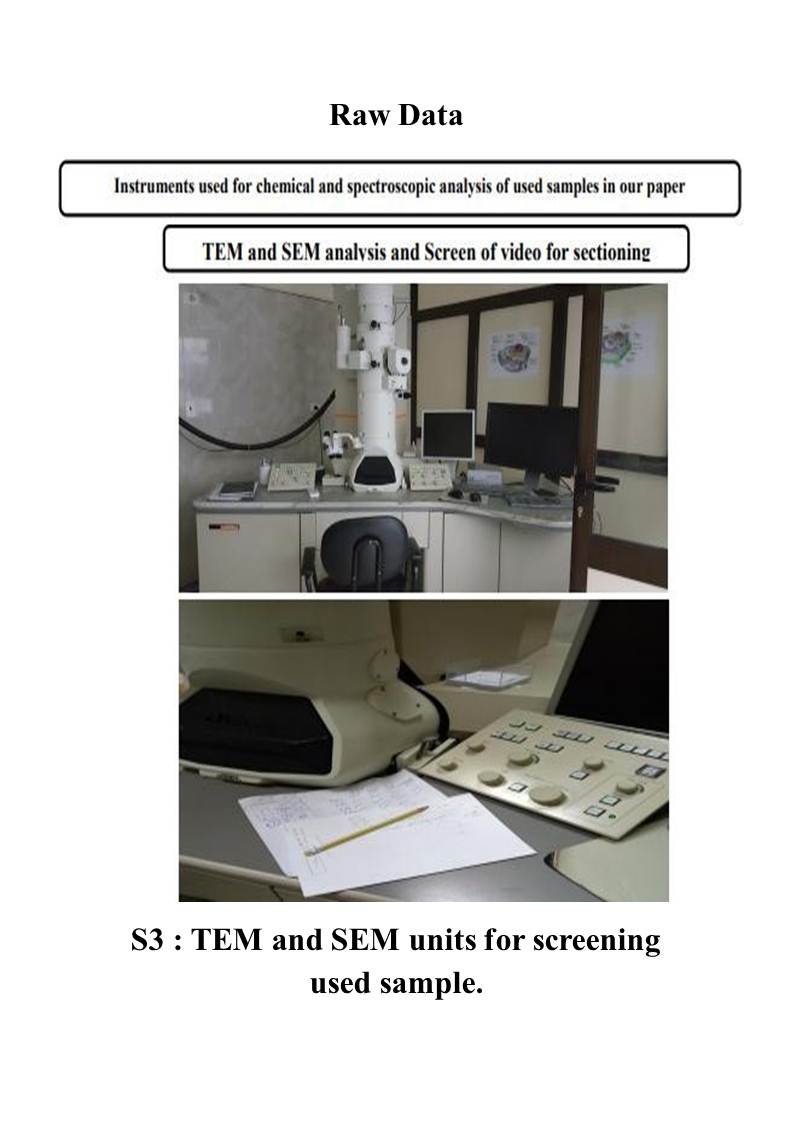

Supplement: Supplemental Information 13 [file peerj-12-15638-s013.jpg]

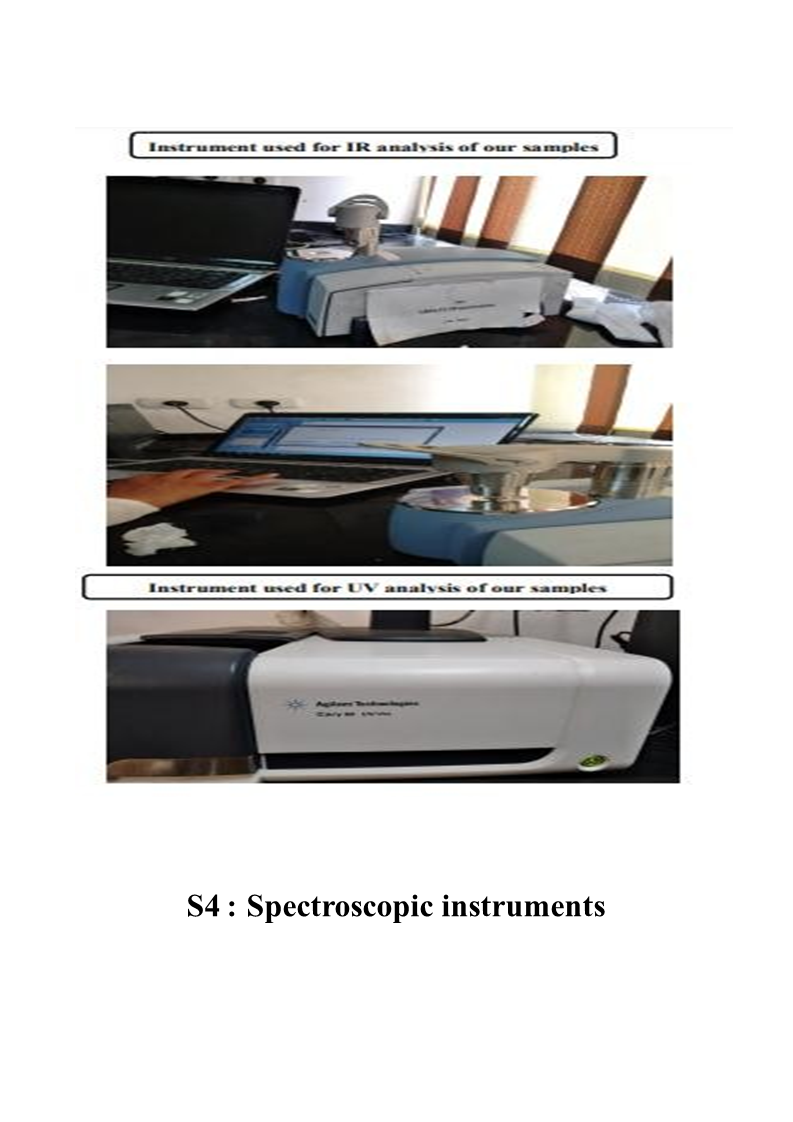

Supplement: Supplemental Information 14 [file peerj-12-15638-s014.png]

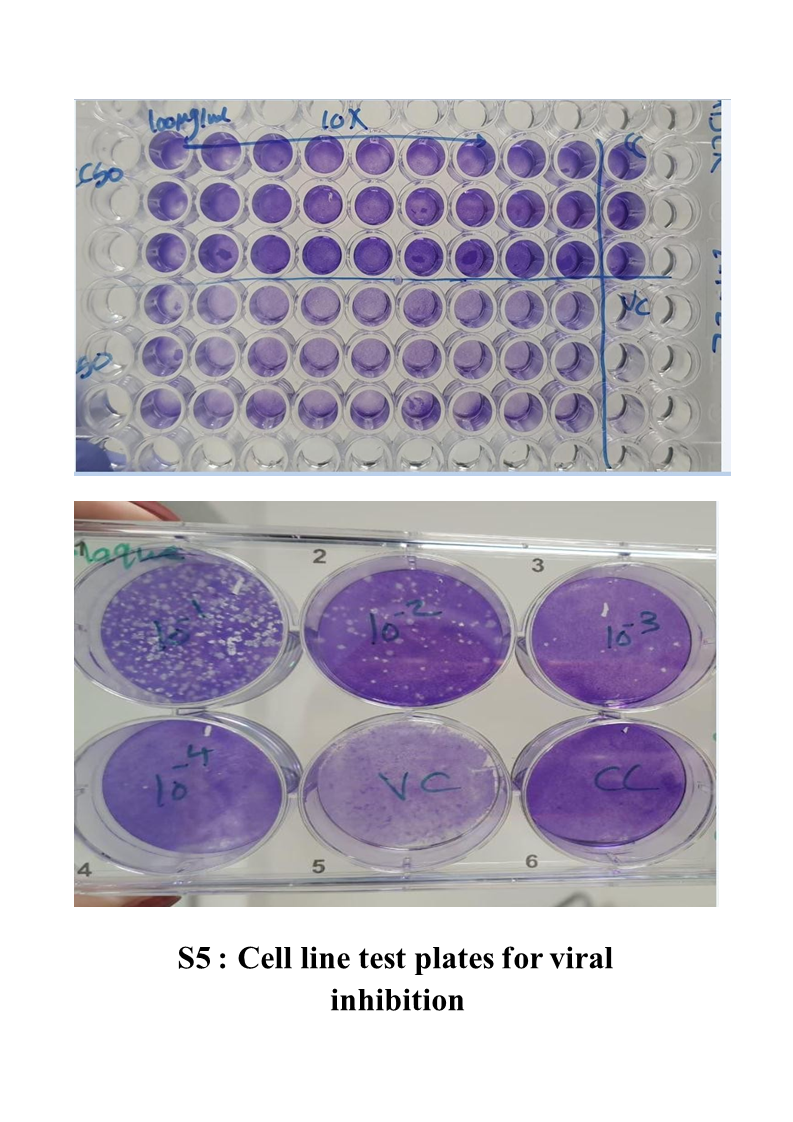

Supplement: Supplemental Information 15 [file peerj-12-15638-s015.png]

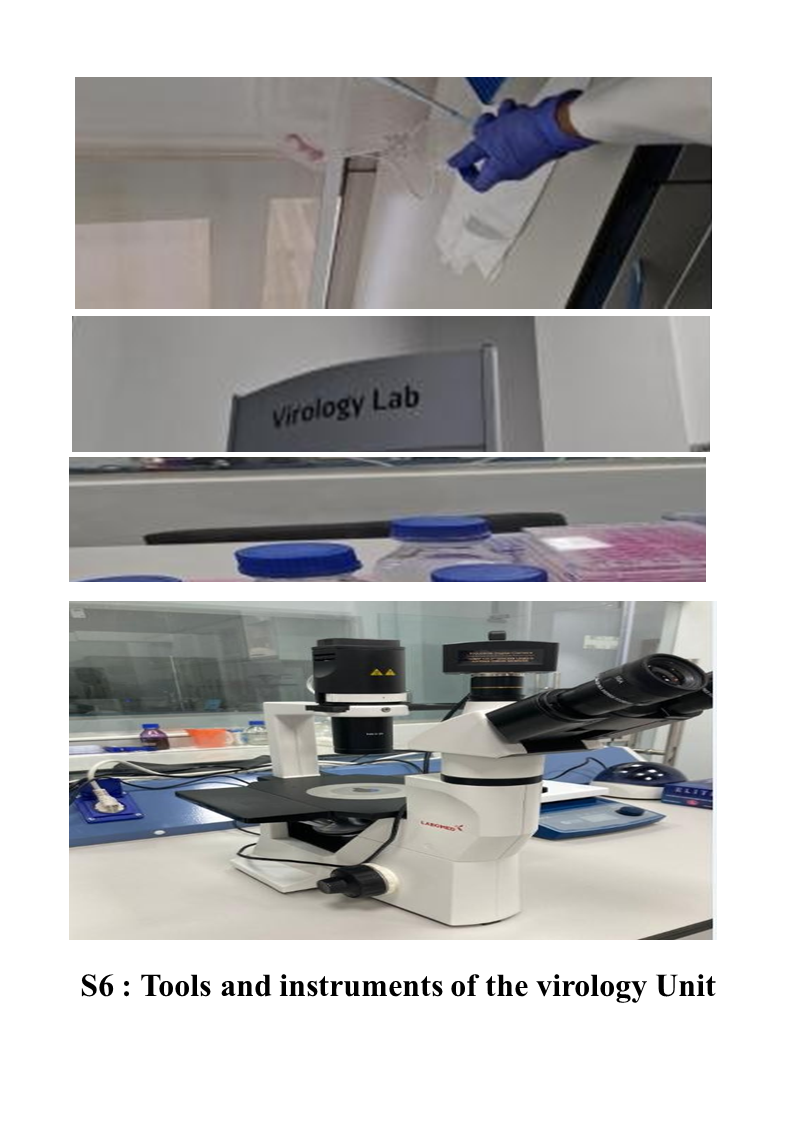

Supplement: Supplemental Information 16 [file peerj-12-15638-s016.png]
