# Supplementary material for: Chemical and spectroscopic characterization of (Artemisinin/Querctin/ Zinc) novel mixed ligand complex with assessment of its potent high antiviral activity against SARS-CoV-2 and antioxidant capacity against toxicity induced by acrylamide in male rats
Source: PeerJ. 2024 Jan 2;12:e15638. doi: 10.7717/peerj.15638 (PMC10768679; doi:10.7717/peerj.15638)
Supplement: Supplemental Information 10 [file peerj-12-15638-s010.pdf]

```
DESCRIPTIVES VARIABLES=Groups ALT AST LDH IL6 TNF CRP
  /STATISTICS=MEAN STDDEV VARIANCE MIN MAX SEMEAN.
```

Descriptives

[DataSet0]

Descriptive Statistics

	N	Minimum	Maximum	Mean		Std. Deviation	Variance
	Statistic	Statistic	Statistic	Statistic	Std. Error	Statistic	Statistic
Groups	20	1.00	4.00	2.5000	.25649	1.14708	1.316
ALT	20	13.02	184.91	59.4200	16.66537	74.52980	5.555E3
AST	20	14.00	294.53	86.6935	27.54274	123.17488	1.517E4
LDH	20	140.43	545.43	2.5670E2	38.61683	172.69973	2.983E4
IL6	20	3.39	49.71	15.8380	4.49326	20.09445	403.787
TNF	20	3.79	62.77	20.4435	5.63851	25.21618	635.856
CRP	19	2.22	96.11	23.6453	8.82588	38.47112	1.480E3
Valid N (listwise)	19						

```
ONEWAY ALT AST LDH IL6 TNF CRP BY Groups
  /STATISTICS DESCRIPTIVES HOMOGENEITY
  /MISSING ANALYSIS
  /POSTHOC=BTUKEY DUNCAN LSD ALPHA(0.05).
```

Oneway

[DataSet0]

Descriptives

		N	Mean	Std. Deviation	Std. Error	95% Confidence Interval for Mean	
						Lower Bound	Upper Bound
ALT	Control group	5	13.1140	.05273	.02358	13.0485	13.1795
	Acy	5	1.8489E2	.01871	.00837	184.8668	184.9132
	Art/Q/Zn	5	13.2280	.01483	.00663	13.2096	13.2464
	Acy + Art/Q/Zn	5	26.4480	.03271	.01463	26.4074	26.4886
	Total	20	59.4200	74.52980	16.66537	24.5390	94.3010
AST	Control group	5	14.1160	.06656	.02977	14.0334	14.1986
	Acy	5	2.9452E2	.01140	.00510	294.5018	294.5302
	Art/Q/Zn	5	14.0460	.02702	.01208	14.0125	14.0795
	Acy + Art/Q/Zn	5	24.0960	.01140	.00510	24.0818	24.1102
	Total	20	86.6935	123.17488	27.54274	29.0459	144.3411
LDH	Control group	5	1.4051E2	.13278	.05938	140.3491	140.6789
	Acy	5	5.4541E2	.01304	.00583	545.3918	545.4242
	Art/Q/Zn	5	1.4212E2	.12696	.05678	141.9604	142.2756
	Acy + Art/Q/Zn	5	1.9878E2	.07893	.03530	198.6780	198.8740
	Total	20	2.5670E2	172.69973	38.61683	175.8780	337.5300
IL6	Control group	5	3.4080	.01643	.00735	3.3876	3.4284
	Acy	5	49.7020	.00837	.00374	49.6916	49.7124
	Art/Q/Zn	5	3.8160	.01140	.00510	3.8018	3.8302
	Acy + Art/Q/Zn	5	6.4260	.01140	.00510	6.4118	6.4402
	Total	20	15.8380	20.09445	4.49326	6.4335	25.2425
TNF	Control group	5	4.4740	.00548	.00245	4.4672	4.4808
	Acy	5	62.7540	.00894	.00400	62.7429	62.7651
	Art/Q/Zn	5	3.8000	.00707	.00316	3.7912	3.8088
	Acy + Art/Q/Zn	5	10.7460	.01817	.00812	10.7234	10.7686
	Total	20	20.4435	25.21618	5.63851	8.6420	32.2450
CRP	Control group	5	2.2340	.00894	.00400	2.2229	2.2451
	Acy	5	96.0960	.01140	.00510	96.0818	96.1102
	Art/Q/Zn	5	4.2340	.00894	.00400	4.2229	4.2451
	Acy + Art/Q/Zn	5	6.5080	.00837	.00374	6.4976	6.5184
	Total	20	27.2680	40.79970	9.12309	8.1732	46.3628

Descriptives

		Minimum	Maximum
ALT	Control group	13.02	13.14
	Acy	184.87	184.91
	Art/Q/Zn	13.21	13.25
	Acy + Art/Q/Zn	26.40	26.49
	Total	13.02	184.91
AST	Control group	14.00	14.16
	Acy	294.50	294.53
	Art/Q/Zn	14.00	14.07
	Acy + Art/Q/Zn	24.08	24.11
	Total	14.00	294.53
LDH	Control group	140.43	140.75
	Acy	545.40	545.43
	Art/Q/Zn	142.00	142.32
	Acy + Art/Q/Zn	198.65	198.86
	Total	140.43	545.43
IL6	Control group	3.39	3.43
	Acy	49.69	49.71
	Art/Q/Zn	3.80	3.83
	Acy + Art/Q/Zn	6.41	6.44
	Total	3.39	49.71
TNF	Control group	4.47	4.48
	Acy	62.75	62.77
	Art/Q/Zn	3.79	3.81
	Acy + Art/Q/Zn	10.72	10.77
	Total	3.79	62.77
CRP	Control group	2.22	2.24
	Acy	96.08	96.11
	Art/Q/Zn	4.22	4.24
	Acy + Art/Q/Zn	6.50	6.52
	Total	2.22	96.11

Test of Homogeneity of Variances

	Levene Statistic	df1	df2	Sig.
ALT	1.755	3	16	.196
AST	2.995	3	16	.062
LDH	1.896	3	16	.171
IL6	1.471	3	16	.260

Test of Homogeneity of Variances

	Levene Statistic	df1	df2	Sig.
TNF	1.722	3	16	.203
CRP	.246	3	16	.863

ANOVA

		Sum of Squares	df	Mean Square	F	Sig.
ALT	Between Groups	105539.101	3	35179.700	3.184E7	.000
	Within Groups	.018	16	.001		
	Total	105539.119	19			
AST	Between Groups	288268.956	3	96089.652	7.091E7	.000
	Within Groups	.022	16	.001		
	Total	288268.978	19			
LDH	Between Groups	566678.601	3	188892.867	1.882E7	.000
	Within Groups	.161	16	.010		
	Total	566678.762	19			
IL6	Between Groups	7671.948	3	2557.316	1.705E7	.000
	Within Groups	.002	16	.000		
	Total	7671.951	19			
TNF	Between Groups	12081.255	3	4027.085	3.287E7	.000
	Within Groups	.002	16	.000		
	Total	12081.257	19			
CRP	Between Groups	31627.687	3	10542.562	1.171E8	.000
	Within Groups	.001	16	.000		
	Total	31627.689	19			

Post Hoc Tests

Multiple Comparisons

Dependent Variable		(I) Groups	(J) Groups	Mean Difference (I-J)	Std. Error	Sig.	95% Confidence Interval	
							Lower Bound	Upper Bound
ALT	LSD	Control group	Acy	-171.77600*	.02102	.000	-171.8206-	-171.7314-
			Art/Q/Zn	-.11400*	.02102	.000	-.1586-	-.0694-
			Acy + Art/Q/Zn	-13.33400*	.02102	.000	-13.3786-	-13.2894-
		Acy	Control group	171.77600*	.02102	.000	171.7314	171.8206
			Art/Q/Zn	171.66200*	.02102	.000	171.6174	171.7066
			Acy + Art/Q/Zn	158.44200*	.02102	.000	158.3974	158.4866

*. The mean difference is significant at the 0.05 level.

Multiple Comparisons

Dependent Variable		(I) Groups	(J) Groups	Mean Difference (I-J)	Std. Error	Sig.	95% Confidence Interval	
							Lower Bound	Upper Bound
ALT	LSD	Art/Q/Zn	Control group	.11400*	.02102	.000	.0694	.1586
			Acy	-171.66200*	.02102	.000	-171.7066-	-171.6174-
			Acy + Art/Q/Zn	-13.22000*	.02102	.000	-13.2646-	-13.1754-
		Acy + Art/Q/Zn	Control group	13.33400*	.02102	.000	13.2894	13.3786
			Acy	-158.44200*	.02102	.000	-158.4866-	-158.3974-
			Art/Q/Zn	13.22000*	.02102	.000	13.1754	13.2646
AST	LSD	Control group	Acy	-280.40000*	.02328	.000	-280.4494-	-280.3506-
			Art/Q/Zn	.07000*	.02328	.008	.0206	.1194
			Acy + Art/Q/Zn	-9.98000*	.02328	.000	-10.0294-	-9.9306-
		Acy	Control group	280.40000*	.02328	.000	280.3506	280.4494
			Art/Q/Zn	280.47000*	.02328	.000	280.4206	280.5194
			Acy + Art/Q/Zn	270.42000*	.02328	.000	270.3706	270.4694
		Art/Q/Zn	Control group	-.07000*	.02328	.008	-.1194-	-.0206-
			Acy	-280.47000*	.02328	.000	-280.5194-	-280.4206-
			Acy + Art/Q/Zn	-10.05000*	.02328	.000	-10.0994-	-10.0006-
		Acy + Art/Q/Zn	Control group	9.98000*	.02328	.000	9.9306	10.0294
			Acy	-270.42000*	.02328	.000	-270.4694-	-270.3706-
			Art/Q/Zn	10.05000*	.02328	.000	10.0006	10.0994
LDH	LSD	Control group	Acy	-404.89400*	.06336	.000	-405.0283-	-404.7597-
			Art/Q/Zn	-1.60400*	.06336	.000	-1.7383-	-1.4697-
			Acy + Art/Q/Zn	-58.26200*	.06336	.000	-58.3963-	-58.1277-
		Acy	Control group	404.89400*	.06336	.000	404.7597	405.0283
			Art/Q/Zn	403.29000*	.06336	.000	403.1557	403.4243
			Acy + Art/Q/Zn	346.63200*	.06336	.000	346.4977	346.7663
		Art/Q/Zn	Control group	1.60400*	.06336	.000	1.4697	1.7383
			Acy	-403.29000*	.06336	.000	-403.4243-	-403.1557-
			Acy + Art/Q/Zn	-56.65800*	.06336	.000	-56.7923-	-56.5237-
		Acy + Art/Q/Zn	Control group	58.26200*	.06336	.000	58.1277	58.3963
			Acy	-346.63200*	.06336	.000	-346.7663-	-346.4977-
			Art/Q/Zn	56.65800*	.06336	.000	56.5237	56.7923
IL6	LSD	Control group	Acy	-46.29400*	.00775	.000	-46.3104-	-46.2776-
			Art/Q/Zn	-.40800*	.00775	.000	-.4244-	-.3916-
			Acy + Art/Q/Zn	-3.01800*	.00775	.000	-3.0344-	-3.0016-
		Acy	Control group	46.29400*	.00775	.000	46.2776	46.3104
			Art/Q/Zn	45.88600*	.00775	.000	45.8696	45.9024
			Acy + Art/Q/Zn	43.27600*	.00775	.000	43.2596	43.2924
		Art/Q/Zn	Control group	.40800*	.00775	.000	.3916	.4244
			Acy	-45.88600*	.00775	.000	-45.9024-	-45.8696-
			Acy + Art/Q/Zn	-2.61000*	.00775	.000	-2.6264-	-2.5936-

*. The mean difference is significant at the 0.05 level.

Multiple Comparisons

Dependent Variable		(I) Groups	(J) Groups	Mean Difference (I-J)	Std. Error	Sig.	95% Confidence Interval	
							Lower Bound	Upper Bound
IL6	LSD	Acy + Art/Q/Zn	Control group	3.01800*	.00775	.000	3.0016	3.0344
			Acy	-43.27600*	.00775	.000	-43.2924-	-43.2596-
			Art/Q/Zn	2.61000*	.00775	.000	2.5936	2.6264
TNF	LSD	Control group	Acy	-58.28000*	.00700	.000	-58.2948-	-58.2652-
			Art/Q/Zn	.67400*	.00700	.000	.6592	.6888
			Acy + Art/Q/Zn	-6.27200*	.00700	.000	-6.2868-	-6.2572-
		Acy	Control group	58.28000*	.00700	.000	58.2652	58.2948
			Art/Q/Zn	58.95400*	.00700	.000	58.9392	58.9688
			Acy + Art/Q/Zn	52.00800*	.00700	.000	51.9932	52.0228
		Art/Q/Zn	Control group	-.67400*	.00700	.000	-.6888-	-.6592-
			Acy	-58.95400*	.00700	.000	-58.9688-	-58.9392-
			Acy + Art/Q/Zn	-6.94600*	.00700	.000	-6.9608-	-6.9312-
		Acy + Art/Q/Zn	Control group	6.27200*	.00700	.000	6.2572	6.2868
			Acy	-52.00800*	.00700	.000	-52.0228-	-51.9932-
			Art/Q/Zn	6.94600*	.00700	.000	6.9312	6.9608
CRP	LSD	Control group	Acy	-93.86200*	.00600	.000	-93.8747-	-93.8493-
			Art/Q/Zn	-2.00000*	.00600	.000	-2.0127-	-1.9873-
			Acy + Art/Q/Zn	-4.27400*	.00600	.000	-4.2867-	-4.2613-
		Acy	Control group	93.86200*	.00600	.000	93.8493	93.8747
			Art/Q/Zn	91.86200*	.00600	.000	91.8493	91.8747
			Acy + Art/Q/Zn	89.58800*	.00600	.000	89.5753	89.6007
		Art/Q/Zn	Control group	2.00000*	.00600	.000	1.9873	2.0127
			Acy	-91.86200*	.00600	.000	-91.8747-	-91.8493-
			Acy + Art/Q/Zn	-2.27400*	.00600	.000	-2.2867-	-2.2613-
		Acy + Art/Q/Zn	Control group	4.27400*	.00600	.000	4.2613	4.2867
			Acy	-89.58800*	.00600	.000	-89.6007-	-89.5753-
			Art/Q/Zn	2.27400*	.00600	.000	2.2613	2.2867

*. The mean difference is significant at the 0.05 level.

Homogeneous Subsets

ALT

Groups		N	Subset for alpha = 0.05			
			1	2	3	4
Tukey …	Control group	5	13.1140			

Means for groups in homogeneous subsets are displayed.

a. Uses Harmonic Mean Sample Size = 5.000.

ALT

	Groups	N	Subset for alpha = 0.05			
			1	2	3	4
Tukey B[a]	Art/Q/Zn	5		13.2280		
	Acy + Art/Q/Zn	5			26.4480	
	Acy	5				1.8489E2
Duncan[a]	Control group	5	13.1140			
	Art/Q/Zn	5		13.2280		
	Acy + Art/Q/Zn	5			26.4480	
	Acy	5				1.8489E2
	Sig.		1.000	1.000	1.000	1.000

Means for groups in homogeneous subsets are displayed.

a. Uses Harmonic Mean Sample Size = 5.000.

AST

	Groups	N	Subset for alpha = 0.05			
			1	2	3	4
Tukey B[a]	Art/Q/Zn	5	14.0460			
	Control group	5		14.1160		
	Acy + Art/Q/Zn	5			24.0960	
	Acy	5				2.9452E2
Duncan[a]	Art/Q/Zn	5	14.0460			
	Control group	5		14.1160		
	Acy + Art/Q/Zn	5			24.0960	
	Acy	5				2.9452E2
	Sig.		1.000	1.000	1.000	1.000

Means for groups in homogeneous subsets are displayed.

a. Uses Harmonic Mean Sample Size = 5.000.

LDH

	Groups	N	Subset for alpha = 0.05			
			1	2	3	4
Tukey B[a]	Control group	5	1.4051E2			
	Art/Q/Zn	5		1.4212E2		
	Acy + Art/Q/Zn	5			1.9878E2	
	Acy	5				5.4541E2
Duncan[a]	Control group	5	1.4051E2			
	Art/Q/Zn	5		1.4212E2		
	Acy + Art/Q/Zn	5			1.9878E2	
	Acy	5				5.4541E2
	Sig.		1.000	1.000	1.000	1.000

Means for groups in homogeneous subsets are displayed.

a. Uses Harmonic Mean Sample Size = 5.000.

IL6

	Groups	N	Subset for alpha = 0.05			
			1	2	3	4
Tukey B[a]	Control group	5	3.4080			
	Art/Q/Zn	5		3.8160		
	Acy + Art/Q/Zn	5			6.4260	
	Acy	5				49.7020
Duncan[a]	Control group	5	3.4080			
	Art/Q/Zn	5		3.8160		
	Acy + Art/Q/Zn	5			6.4260	
	Acy	5				49.7020
	Sig.		1.000	1.000	1.000	1.000

Means for groups in homogeneous subsets are displayed.

a. Uses Harmonic Mean Sample Size = 5.000.

TNF

	Groups	N	Subset for alpha = 0.05			
			1	2	3	4
Tukey B[a]	Art/Q/Zn	5	3.8000			
	Control group	5		4.4740		
	Acy + Art/Q/Zn	5			10.7460	
	Acy	5				62.7540
Duncan[a]	Art/Q/Zn	5	3.8000			
	Control group	5		4.4740		
	Acy + Art/Q/Zn	5			10.7460	
	Acy	5				62.7540
	Sig.		1.000	1.000	1.000	1.000

Means for groups in homogeneous subsets are displayed.

a. Uses Harmonic Mean Sample Size = 5.000.

CRP

	Groups	N	Subset for alpha = 0.05			
			1	2	3	4
Tukey B[a]	Control group	5	2.2340			
	Art/Q/Zn	5		4.2340		
	Acy + Art/Q/Zn	5			6.5080	
	Acy	5				96.0960
Duncan[a]	Control group	5	2.2340			
	Art/Q/Zn	5		4.2340		
	Acy + Art/Q/Zn	5			6.5080	

Means for groups in homogeneous subsets are displayed.

a. Uses Harmonic Mean Sample Size = 5.000.

CRP

	Groups	N	Subset for alpha = 0.05			
			1	2	3	4
Duncan[a]	Acy	5				96.0960
	Sig.		1.000	1.000	1.000	1.000

Means for groups in homogeneous subsets are displayed.

a. Uses Harmonic Mean Sample Size = 5.000.
